# Radiation-induced G_1_ arrest is not defective in fibroblasts from Li-Fraumeni families without *TP53* mutations

**DOI:** 10.1038/sj.bjc.6690265

**Published:** 1999-04

**Authors:** J M Boyle, M J Greaves, R S Camplejohn, J M Birch, S A Roberts, J M Varley

**Affiliations:** CRC Sections of Molecular Genetics, Biomathematics and Computing, Paterson Institute for Cancer Research, Christie CRC Research Centre, Manchester, M20 4BX, UK; Richard Dimbleby Department of Cancer Research, UMDS, St. Thomas's Hospital, London, SE1 7EH, UK; CRC Paediatric and Familial Cancer Research Group, Royal Manchester Children's Hospital, Manchester, M27 4HA, UK

**Keywords:** Li-Fraumeni syndrome, G_1_ arrest, cell cycle, fibroblasts, radiosensitivity, p53

## Abstract

Radiation-induced G_1_ arrest was studied in four classes of early passage skin fibroblasts comprising 12 normals, 12 heterozygous (mut/wt) *TP53* mutation-carriers, two homozygous (mut/–) *TP53* mutation-carriers and 16 strains from nine Li-Fraumeni syndrome or Li-Fraumeni-like families in which no *TP53* mutation has been found, despite sequencing of all exons, exon–intron boundaries, 3′ and 5′ untranslated regions and promoter regions. In an assay of p53 allelic expression in yeast, cDNAs from these non-mutation strains behaved as wild-type p53. Using two different assays, we found G_1_ arrest was reduced in heterozygous strains with mis-sense mutations and one truncation mutation, when compared to the range established for the normal cells. Heterozygous strains with mutations at splice sites behaved like normal cells, whilst homozygous (mut/–) strains showed either extremely reduced, or no, arrest. Strains from all nine non-mutation families gave responses within the normal range. Exceptions to the previously reported inverse correlation between G_1_ arrest and clonogenic radiation resistance were observed, indicating that these phenotypes are not strictly interdependent. © 1999 Cancer Research Campaign


*British Journal of Cancer* (1999) **79**(11/12), 1657–1664
© 1999 Cancer Research Campaign
Article no. bjoc.1998.0265

### Radiation-induced $G_1$ arrest is not defective in fibroblasts from Li-Fraumeni families without *TP53* mutations

**JM Boyle[1], MJ Greaves[1], RS Camplejohn[2], JM Birch[3], SA Roberts[4] and JM Varley[1]**

CRC Sections of [1]Molecular Genetics and [4]Biomathematics and Computing, Paterson Institute for Cancer Research, Christie CRC Research Centre, Manchester M20 4BX, UK; [2]Richard Dimbleby Department of Cancer Research, UMDS, St. Thomas's Hospital, London SE1 7EH, UK; [3]CRC Paediatric and Familial Cancer Research Group, Royal Manchester Children's Hospital, Manchester M27 4HA, UK

**Summary** Radiation-induced $G_1$ arrest was studied in four classes of early passage skin fibroblasts comprising 12 normals, 12 heterozygous (mut/wt) *TP53* mutation-carriers, two homozygous (mut/–) *TP53* mutation-carriers and 16 strains from nine Li-Fraumeni syndrome or Li-Fraumeni-like families in which no *TP53* mutation has been found, despite sequencing of all exons, exon–intron boundaries, 3′ and 5′ untranslated regions and promoter regions. In an assay of p53 allelic expression in yeast, cDNAs from these non-mutation strains behaved as wild-type p53. Using two different assays, we found $G_1$ arrest was reduced in heterozygous strains with mis-sense mutations and one truncation mutation, when compared to the range established for the normal cells. Heterozygous strains with mutations at splice sites behaved like normal cells, whilst homozygous (mut/–) strains showed either extremely reduced, or no, arrest. Strains from all nine non-mutation families gave responses within the normal range. Exceptions to the previously reported inverse correlation between $G_1$ arrest and clonogenic radiation resistance were observed, indicating that these phenotypes are not strictly interdependent.

**Keywords:** Li-Fraumeni syndrome; $G_1$ arrest; cell cycle; fibroblasts; radiosensitivity; p53

Classical Li-Fraumeni syndrome (LFS) families have a proband with sarcoma under the age of 45 years, a first-degree relative with any cancer under age 45, and a first-or second-degree relative with either a sarcoma at any age or any other cancer under age 45 years (Li and Fraumeni, 1969; Li et al, 1988). Li-Fraumeni-like (LFL) families conform to a broader definition (Birch et al, 1994) with a proband having any childhood cancer or sarcoma, brain tumour or adrenal cortical tumour diagnosed before age 45 years with one first- or second-degree relative with a typical syndromal cancer at any age, plus a first- or second-degree relative with any cancer under age 60 years. In both syndromes the predominant cancers are bone and soft tissue sarcomas and breast cancer, plus an excess of brain tumours, leukaemia and adrenocortical carcinomas diagnosed under age 45 years. We have reported germline mutations in the tumour suppressor gene, *TP53*, in 14 of 21 LFS families and four of 18 LFL families (Birch et al, 1994; Varley et al, 1997). An understanding of the consequences of the p53 mutations, particularly in the mesenchymal cells, could have implications for the management and counselling of these families.

Pioneering work by Little (1968, 1970) demonstrated that human cells arrest in the $G_1$ phase of the cell cycle following exposure to ionizing radiation. Subsequently, it was shown that radiation-sensitive fibroblasts from patients with the cancer predisposition syndrome, ataxia-telangiectasia (AT), did not show this response (Little and Nagasawa 1985; Nagasawa et al, 1985). These observations can be explained by the fact that radiation-induced stabilization of p53 expression in AT cells is both delayed and is reduced compared to normal cells, resulting in lower levels of transactivation of the cyclin-dependent protein kinase inhibitor, $p21^{WAF1/CIP1}$, which has been strongly implicated in the permanent arrest of cells in $G_1$ (Di Leonardo et al, 1994; Dulic et al, 1994).

Recently, we reported that radiation-resistant fibroblasts from Li-Fraumeni (LF) individuals also show reduced $G_1$ arrest (Williams et al, 1997). We now report an enlarged study of $G_1$ arrest in which we have compared the variability in responses of 12 fibroblast strains from normal volunteers with that of 30 strains from 20 classic LFS or LFL families. We have used this material to answer the following questions:

1. Permanent $G_1$ arrest has been reported following irradiation of cells synchronized in $G_1$ (Little, 1968, 1970) and transient arrest has been reported following irradiation of asynchronous cells (Kastan et al, 1991). Li et al (1995) questioned whether the two conditions gave the same measure of $G_1$ arrest, but did not answer the question directly. We have directly compared the two methods using the enlarged panel of cells.
2. Do different *TP53* mutations produce different $G_1$ arrest responses?
3. How does loss of the wild-type (wt) allele affect the response?
4. What is the response in cells from LF families in which no *TP53* mutation has been found despite exhaustive sequencing of all exons, exon–intron boundaries, 3′ and 5′ untranslated regions and the promoter region (Varley et al, 1997)?
5. To what extent is reduced $G_1$ arrest a predictor of clonogenic radioresistance?

*Received 16 June 1998*
*Revised 26 August 1998*
*Accepted 27 August 1998*

*Correspondence to:* JM Boyle, CRC Section of Molecular Genetics, Paterson Institute for Cancer Research, Christie CRC Research Centre, Wilmslow Road, Withington, Manchester M20 4BX, UK

**Table 1** Donor details, allelic expression and the magnitude of radiation-induced $G_1$ arrest in fibroblast cultures

| Strains | [a]Family (person) | Type | Mutation | Age at biopsy | Sex | [b]FASAY | [d]Transient arrest | | | [e]Permanent arrest | |
|---|---|---|---|---|---|---|---|---|---|---|---|
| | | | | | | | [c]*n* | 0.5 Gy | 4 Gy | [c]*n* | 4 Gy |
| Group 1 – Normal controls | | | | | | | | | | | |
| 83MA | | | | 34 | F | | 1 | 62.6 | 90.9 | 2 | 85.1 ± 4.0 |
| 85MA | | | | 28 | M | 4 (wt/wt) | 1 | 66.6 | 92.5 | 2 | 50.2 ± 0.8 |
| 93MA | | | | 19 | M | 4 (wt/wt) | 1 | 61.5 | 89.7 | 1 | 67.4 |
| 105MA | | | | 58 | M | | 1 | 67.8 | 95.7 | 1 | 74.1 |
| 120MA | | | | 47 | F | | 1 | 50.0 | 86.4 | 2 | 74.7 ± 7.4 |
| 136MA | 83 (I-1) | [f]Prior to | | 77 | M | | 1 | 54.2 | 81.4 | 1 | 62.1 |
| 141MA | 83 (I-2) | mutation | | 77 | F | | 1 | – | 85.7 | 1 | 76.6 |
| 156MA | | | | 35 | F | | 6 | 57.1 ± 3.9 | 91.0 ± 6.0 | 1 | 66.1 |
| 162MA | | | | 27 | F | | 1 | 53.8 | 90.3 | 1 | 69.5 |
| 176MA | | | | 31 | F | | 8 | 55.4 ± 10.3 | 94.3 ± 1.8 | 2 | 71.8 ± 1.9 |
| 177MA | 2635 (III-1) | [g]Spouse | | 68 | M | 4;4 (wt/wt) | 1 | 72.6 | 97.1 | 2 | 80.3 ± 2.8 |
| 187MA | 86 | [g]Spouse | | 42 | M | | 1 | 59.4 | 93.0 | 1 | 90.9 |
| Group 2 – Mutation-carrier families (mut/wt strains) | | | | | | | | | | | |
| FH1 | 266 (II-4) | LFS aff | R248W/wt | 20 | M | | 1 | 12.5 | 54.6 | 3 | 29.2 ± 7.1 |
| 163MA | 266 (II-2) | LFS aff | R248W/wt | 31 | F | | 1 | 10.2 | 59.3 | 1 | 27.5 |
| 131MA | 222 (IV-1) | LFS aff | R248Q/wt | 18 | M | | 1 | 24.5 | 25.5 | 1 | 25.2 |
| 138MA | 83 (III-4) | LFS unaff | R175H/wt | 16 | M | 45 (mut/wt) | 1 | 28.1 | 73.5 | 1 | 39.2 |
| 124MA | 16 (IV-1) | LFS aff | Y220C/wt | 13 | F | 65 (mut/wt) | 1 | 12.5 | 45.8 | 3 | 14.9 ± 7.0 |
| 109MA | 85 (III-7) | LFL unaff | E180K/wt | 52 | M | 49 (mut/wt) | 1 | 35.6 | 84.2 | 2 | 57.1 ± 4.6 |
| 110MA | 85 (IV-1) | LFL aff | E180K/wt | 18 | F | 58 (mut/wt) | 2 | 43.0 ± 1.6 | 84.7 ± 5.2 | 3 | 51.3 ± 10.7 |
| 168MA | 1502 (III-1) | LFL aff | R273H/wt | 31 | F | | 1 | 28.7 | 69.1 | – | |
| 178MA | 2635 (IV-8) | LFL aff | spl.donor exon 4/wt | 39 | F | 3 (wt/wt) | 1 | 67.5 | 93.5 | 4 | 63.5 ± 10.7 |
| 191MA | 86 (V-5) | LFS aff | spl. acc. intron 3/wt | 16 | M | | 1 | 52.1 | 88.2 | – | |
| 193MA | 2612 (III-1) | LFS aff | R209stop/wt | 41 | M | 12,12 (wt/wt) | 1 | 43.8 | 75.2 | 1 | 44.8 |
| 194MA | 64 (III-5) | LFS unaff | P152L/wt | 42 | F | 57,56 (mut/wt) | 1 | 3.0 | 59.8 | 1 | 54.6 |
| Group 3 – Mutation-carrier families (mut/–strains) | | | | | | | | | | | |
| 161MA-F | 7379 (III-6) | LFS aff | L344P/- | 45 | M | 97 (mut/–) | 1 | –7.1 ± 12.8 | –3.7 ± 7.8 | 1 | 14.1 |
| 172MA | [i] | LFS aff | R337C/- | 34 | F | | 1 | –17.7 | –1.9 | 1 | 19.5 |
| Group 4 – Non-mutation carriers | | | | | | | | | | | |
| 140MA | 22 (III-4) | LFS aff | | 14 | F | 5 (wt/wt) | 1 | 60.5 | 72.2 | 3 | 78.6 ± 11.3 |
| 146MA | 80 (IV-19) | LFL aff | | 65 | F | 4 (wt/wt) | 1 | 47.8 | 61.2 | 1 | 67.3 |
| 147MA | 80 (V-4) | LFL aff | | 42 | F | 4 (wt/wt) | 1 | 76.1 | 92.5 | 4 | 72.8 ± 10.1 |
| 148MA | 80 (V-5) | LFL unaff | | 38 | M | | 1 | 62.4 | 94.1 | 1 | 88.4 |
| 154MA | 80 (V-6) | LFL aff | | 36 | F | | 1 | 62.0 | 96.1 | 1 | 56.1 |
| 79MA | 81 (III-5) | LFS aff | | 70 | F | 9 (wt/wt) | 1 | 70.0 | 96.5 | 2 | 72.5 ± 0.5 |
| 80MA | 81 (IV-1) | LFS aff | | 45 | M | 12;9;4 (wt/wt) | 1 | 64.6 | 94.6 | 1 | 58.7 |
| 81MA | 81 (IV-3) | LFS unaff | | 40 | F | 9 (wt/wt) | 1 | 47.6 | 90.6 | 3 | 56.4 ± 2.1 |
| 115MA | 82 (IV-5) | LFS aff | | 21 | M | | – | – | – | 1 | 70.0 |
| 126MA | 88 (II-2) | LFS aff | | 29 | M | 5 (wt/wt) | 2 | 69.1 ± 9.8 | 93.8 ± 0.4 | 2 | 74.4 ± 0.5 |
| 130MA | 88 (I-1) | LFS unaff | | 59 | M | | 1 | 28.0 | 71.0 | 2 | 85.1 ± 8.0 |
| 121MA | 119 (II-2) | LFS aff | | 43 | M | 8 (wt/wt) | 1 | 45.6 | 75.0 | 1 | 75.2 |
| 122MA | 119 (II-1) | LFS aff | | 46 | M | | 1 | 47.4 | 76.3 | 1 | 73.2 |
| 127MA | 338 (III-2) | LFL aff | | 38 | F | 7;7 (wt/wt) | 1 | 58.8 | 92.6 | 2 | 78.9 ± 4.9 |
| 128MA | 348 (III-2) | LFL unaff | | 43 | F | 26;15;8(wt/wt) | 1 | 65.5 | 95.7 | 1 | 79.4 |
| 107MA | 353 (III-3) | LFL aff | | 34 | F | 6 (wt/wt) | 1 | 45.2 | 90.3 | 1 | 77.5 |

[a]Family and person numbers as listed in Varley et al (1997); [b]% red (mutant) colonies obtained from separate cDNA preparations with the inferred allelic expression in parentheses; [c]*n*, number of determinations; [d]Values are % inhibition of S phase; [e]Values are % reduction of labelled cells; [f]Parents of de novo mutation; [g]Uninvolved spouse; [h]Cancer affected (aff) or unaffected (unaff) at time of biopsy; [i]Barnes et al (1992). M, male; F, female.

### MATERIALS AND METHODS

### Fibroblast strains and clonogenic survival

Most fibroblast strains used here and their culture conditions have been described in detail (Boyle et al, 1998; Varley et al, 1998). The origins of additional strains are given in Table 1. Early passage cultures were used in the experiments reported. Clonogenic survival following exposure to low-dose rate (0.011 Gy per min) $^{60}$Co radiation was measured as previously described (Boyle et al, 1998).

### Measurement of $G_1$ arrest

Radiation-induced $G_1$ arrest was determined by two methods. Transient arrest was determined essentially as described by Kuerbitz et al (1992). Confluent cultures were subcultured by 1:3 dilution approximately 3 days before trypsinization. Asynchronous cell suspensions at $1 \times 10^6$ per ml were irradiated at room temperature with 0, 0.5 or 4 Gy $^{137}$Cs gamma radiation at a dose rate of 3.3 Gy per min, then 1 ml aliquots were inoculated into T150 flasks (Falcon) and incubated at 37°C for 17 h. BrdU was added to 10 μM

 *© Cancer Research Campaign 1999*

and incubation was continued for 4 h, when the cells were harvested by trypsinization, washed in phosphate buffered saline (PBS), resuspended and fixed in 70% ethanol. Fixed suspensions were stored at –20°C prior to staining for FACS analysis. Suspensions were treated at room temperature with 2 N hydrochloric acid for 30 min, then neutralized with borate buffer. Cells were stained by sequential adsorption of mouse anti-BrdU monoclonal antibody, clone BU20a, and rabbit anti-mouse antibody conjugated to fluorescein isothiocyanate (FITC; both from Dako A/S, Denmark). Incorporation of BrdU was analysed by FACS on a FACSCAN using Cellquest (Becton-Dickinson) software.

Permanent $G_1$ arrest was determined according to Williams et al (1997). Monolayer cultures were held confluent for 10–14 days prior to 48 h incubation in medium containing 0.1% fetal calf serum. The $G_1$ synchronized monolayers were irradiated with 0 or 4 Gy $^{137}$Cs gamma radiation (3.3 Gy per min), trypsinized and plated at $4 \times 10^4$ cells per 3 cm diameter petri dish with 1 μCi [$^3$H]-TdR (20 Ci per mM; New England Nuclear). Five plates at each dose were fixed at intervals between 60 and 120 h post-irradiation incubation, the period corresponding to the maximum cumulative labelling index. Plates were coated with Ilford K5 photographic emulsion and stored at 4°C for about 2 weeks prior to developing and staining with Giemsa. The mean fraction of cells with labelled nuclei was determined for each set of five plates and the percentage of inhibition of S phase was calculated by comparing the labelled fractions of the irradiated and unirradiated cells.

### Functional assay of alleles in yeast (FASAY)

Messenger RNA was extracted from early passage cells and cDNA was derived and used to test the ability of p53 to transactivate a target gene as previously described (Flaman et al, 1995; Lomax et al, 1997).

### Statistical analysis

Statistical analysis was performed using the SPSS statistics package. Fibroblast strains were grouped as normal, LF with mutation and LF without mutation, according to their origin and *TP53* status. $G_1$ arrest was analysed using the Mann–Whitney *U*-test to

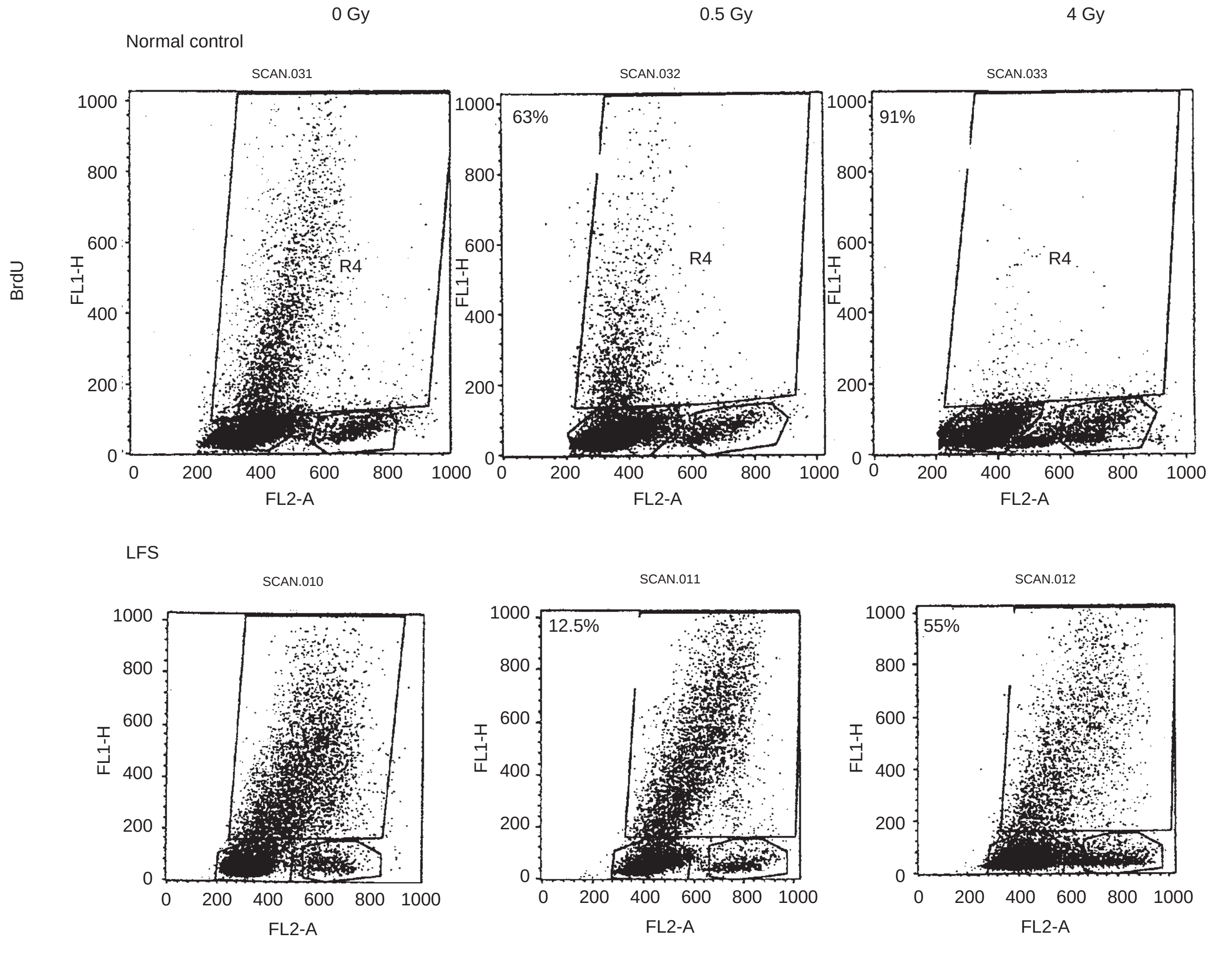

**Figure 1** FACS profiles of BrdU labelled cells. Cells were irradiated as indicated and labelled with BrdU under conditions of transient arrest (Materials and methods). Panels show the bivariate distributions and gating of unlabelled G1 and G2 cells and the cells that were in S phase at the time of labelling. Values show the percentage inhibition of S phase in a normal control (83MA) and an LFS strain (FH1)

© Cancer Research Campaign 1999

compare the group means of the aggregated data of all assays. Transient and permanent arrest data were compared using Spearman's rank correlation.

### RESULTS

Experiments were performed on four groups of cells represented by 12 strains derived from normal individuals, 12 *TP53* heterozygous strains from LFS and LFL families, two strains with *TP53* mutations that had lost the wild-type allele and 16 strains derived from nine non-mutation families (Table 1).

### Allelic expression in yeast

As an additional test for the absence of *TP53* mutations in cells derived from families in which no mutations had been detected by sequencing, fibroblast-derived cDNA was used to test the ability of p53 to transactivate a target gene in yeast cells. Fibroblasts from classic LFS families having mis-sense mutations in the DNA binding region of p53 were used as positive controls (e.g. codons 180, 220) and resulted in an approximately 1:1 ratio of mutant:wt colonies (49–65% mutant; Table 1). Strain 161MA-F, which showed loss of the wild-type allele (mut/–) gave virtually 100% red (mutant) colonies. Cells from normal individuals acted as negative controls and yielded a background frequency of red colonies of < 10%. A mutation affecting splicing (178MA), and a mutation in codon 209 converting it to a stop codon (193MA), were not detected as mutant in the transactivation assay. We used the assay to test p53 expression in fibroblasts from each of the nine LF families in which no *TP53* mutations had been found. In no case was a 1:1 ratio of colonies observed and seven families gave < 10% red colonies indicative of wt p53. However, cells from two families, represented by 80MA and 128MA, consistently gave somewhat elevated frequencies of red colonies, but not approaching a 1:1 ratio. Also, when compared with our experience of normal cells or mis-sense LFS, there was greater variability in the range of values obtained with different cDNA preparations of these cases.

### $G_1$ checkpoint

The effect of mutations in the p53 gene on the $G_1$ checkpoint was determined by comparing the proportion of cells in S phase following $^{137}$Cs radiation. Two independent sets of data were acquired: transient arrest in asynchronous cells was determined by FACS analysis of BrdU incorporation, permanent arrest in cells synchronized in $G_1$ at the time of irradiation was determined by autoradiography of [$^3$H]TdR uptake.

#### *Transient arrest*

Figure 1 shows examples of the DNA profiles and gating of the FACS analyses of a normal control (83MA), and LFS strain FH1 (R248W/wt). The percentage reduction of BrdU-labelled cells occurring as a result of prior exposure to 0.5 and 4 Gy radiation are listed for all strains tested in Table 1. Multiple repeats were performed on two normal strains (156MA and 176MA), otherwise single determinations were performed on most strains.

#### *Permanent arrest*

Table 1 also shows the radiation-induced reduction of labelled cells due to permanent arrest in the $G_1$ phase. The data shown are a combination of those previously reported (Williams et al, 1997) and new data acquired under the same conditions in this study.

#### *Statistical analysis of $G_1$ arrest data*

Comparison of transient and permanent arrest data obtained for all strains after 4 Gy showed a significant correlation (Spearman's correlation coefficient = 0.768, significant at the 0.01 level). However, the significance was principally due to the strong correlation correlation (Spearman's correlation coefficient = 0.879,

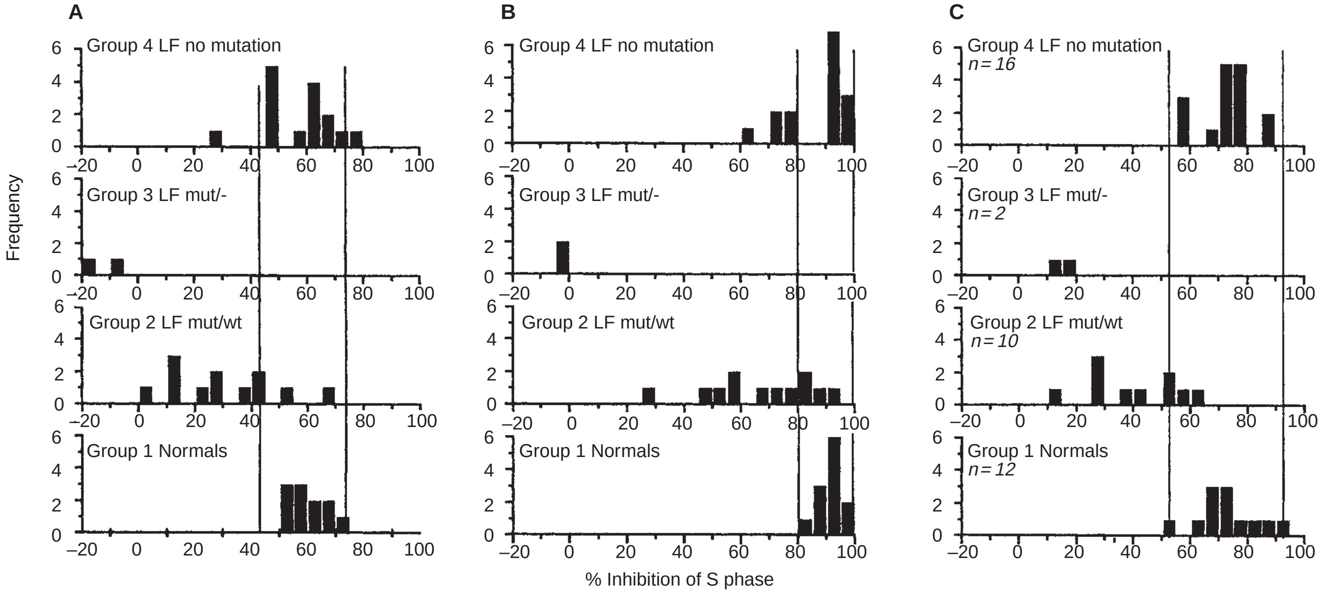

**Figure 2** Histograms of inhibition of S phase under conditions of transient arrest after 0.5 Gy (**A**) and 4 Gy (**B**) or permanent arrest after 4 Gy (**C**). Cells are grouped according to *TP53* status. Mean values from Table 1 were binned at 5% intervals and vertical lines indicate 2 standard deviation limits of the mean values of normal control group 1

 *© Cancer Research Campaign 1999*

**Table 2** Analysis of $G_1$ arrest data

**Summary of statistics**

| Group | Transient arrest | | | | | | Permanent arrest | | |
|---|---|---|---|---|---|---|---|---|---|
| | 0.5 Gy | | | 4 Gy | | | 4 Gy | | |
| | [a]*n* | [b]Mean | SD | *n* | Mean | SD | *n* | Mean | SD |
| 1. Normals | 11 | 60.09 | 6.88 | 12 | 90.67 | 4.46 | 12 | 72.25 | 10.61 |
| 2. LF, wt/mut | 12 | 30.13 | 19.20 | 12 | 67.79 | 19.78 | 10 | 40.74 | 16.07 |
| 3. LF mut/– | 2 | –12.40 | 7.49 | 2 | –2.80 | 1.27 | 2 | 16.80 | 3.82 |
| 4. LF, no mutation | 15 | 46.21 | 22.97 | 15 | 77.76 | 24.31 | 16 | 72.79 | 9.41 |

***P*-values of Mann–Whitney *U*-tests**

| Groups compared | Transient arrest | | Permanent arrest |
|---|---|---|---|
| | 0.5 Gy | 4 Gy | 4 Gy |
| 1 v 2 | 0.001 | 0.001 | < 0.001 |
| 1 v 4 | 0.59 | 0.83 | 0.69 |
| 2 v 4 | 0.001 | 0.005 | < 0.001 |

[a]Number of strains in group; [b]Mean of aggregated means of percentage inhibition of S phase.

significant at the 0.001 level) found for the mut/wt strains (Group 2, Table 1). These data were linearly correlated over a wide range from approximately 20% to 90%. No significant correlations were found for groups 1 and 4 (normals and non-mutants) whose data points lay mainly at the top end of the linear response, clustered between approximately 60–90%.

Variability in the results obtained with the four groups of cells tested is shown graphically in Figure 2. Mann–Whitney *U*-tests were used to compare the group means (Table 2). $G_1$ arrest, both transient and permanent, was significantly reduced in heterozygous LF cells when compared to both normal and non-mutation LF cells. In contrast, there was no significant difference between normal and non-mutant LF cells in either of the assays.

### *Correlations between $G_1$ arrest and clonogenic radiosensitivity*

The relative clonogenic survivals following exposure to 3 and 6 Gy of low-dose rate $^{60}$Co radiation were 34.4 ± 7.1 and 19.5 ± 10.4 for 193MA, and 45.3 ± 16.5 and 17.9 ± 4.5 for 194MA (mean percentage survivals ± 1 standard deviation of three independent experiments). Data for the majority of other strains have been reported previously (Boyle et al, 1998; Varley et al, 1998).

From a clinical perspective it may be important to identify abnormal responses for different endpoints in cells from different individuals. As a general strategy, this can be done by assigning as abnormal those responses that fall outside limits set by 2 standard deviations of the mean values of responses obtained from a panel of normal cells. We used this strategy to assess the effects of *TP53* status on $G_1$ arrest and clonogenic radiosensitivity (Table 3). We defined strains as having compromised $G_1$ arrest if at least two of the three conditions (transient arrest after 0.5 or 4 Gy and permanent arrest after 4 Gy) showed reduced arrest. We defined as radioresistant any strain whose clonogenic survival was greater than the normal limits (41.8% or 14.0%) after exposure to either 3 Gy or 6 Gy, respectively (Boyle et al, 1998).

The effect of *TP53* status on the relationship between $G_1$ arrest and clonogenic radiosensitivity, is shown in 2 × 2 concordance tables (Table 4), from which it is apparent that there are several discordant strains in each LF group. Thus both mut/– strains (161MA-F, 172MA) have reduced $G_1$ arrest but normal radiosensitivity. The dissociation of reduced $G_1$ arrest and radioresistance was also seen in some heterozygous strains and some strains without p53 mutations.

Among heterozygous strains, there were two discordant strains, 110MA (E180K/wt) which had normal $G_1$ arrest but was radioresistant, and 131MA (R248Q/wt) which had reduced $G_1$ arrest and normal radiosensitivity. However, two of ten mutation carrying strains behaved like normal cells in their response to ionizing radiation, having normal $G_1$ arrest and radiosensitivity, and six had reduced $G_1$ arrest and were radioresistant, thus conforming to our original concordance (Table 4).

Of six non-mutation strains tested for both parameters, three (80MA, 81MA, 126MA) were discordant and had normal $G_1$ responses associated with borderline survival responses (Table 3). The three concordant strains, 146MA, 154MA and 79MA, had normal $G_1$ and survival responses. Within family 81, all three strains tested had normal $G_1$ response, but 80MA and 81MA were slightly more resistant than 79MA and were differently categorized.

### DISCUSSION

### Equivalence of $G_1$ methodologies

The two methods that we used to determine radiation-induced $G_1$ arrest in human fibroblast cells showed a highly significant correlation for data from heterozygous LF cells with *TP53* mutations (mut/wt) cells, for which there was a linear response over a wide range of values after 4 Gy exposure. However, permanent $G_1$ arrest appears to be more reliable than transient arrest as a means of discriminating between mut/wt cells and those from families with no mutation (Table 3). Exposure to doses less than 4 Gy may improve the correlation between the two methods for normal and non-mutation groups by inducing less than saturating responses in these cells.

© Cancer Research Campaign 1999 

**Table 3** Comparison of $G_1$ arrest and clonogenic radiosensitivity in LF cells compared to normals

| Strain (family) *TP53* status | [a]$G_1$ Arrest compared to normal controls: Transient 0.5 Gy | Transient 4 Gy | Permanent 4 Gy | [b]Cell survival following 3 Gy/6 Gy |
|---|---|---|---|---|
| Group 2 – Mutation carriers | | | | |
| FH1 (266) R248W/+ | r | r | r | R/R |
| 163MA (266) R248W/+ | r | r | r | R/R |
| 131MA (222) R248Q/+ | r | r | r | n/n |
| 138MA (83) R175H/+ | r | r | r | R/R |
| 124MA (16) Y220C/+ | r | r | r | R/R |
| 109MA (85) E180K/+ | r | n | n | n/n |
| 110MA (85) E180K/+ | r | n | n (0.3) | R/R |
| 168MA (1502) R273H/+ | r | r | nd | nd |
| 178MA (2635) spl. Don. Exon 3/+ | n | n | n | n/n |
| 191MA (86) spl. Acc. Intron 3/- | n | n | nd | nd |
| 193MA (2612) R209stop/+ | r | r | r | n/R |
| 194MA (64) P152L/+ | r | r | n | R/R |
| Group 3 – Mut/- strains | | | | |
| 161MA-F (7379) L344P/- | no arrest | no arrest | r | n/n |
| 172MA R337/- | no arrest | no arrest | r | n/n |
| Group 4 – Non-mutation families | | | | |
| 140MA (22) | n | r | n | nd |
| 146MA (80) | n (1.6) | r | n | n/n |
| 147MA (80) | n | n | n | nd |
| 148MA (80) | n | n | n | nd |
| 154MA (80) | n | n | n | n/n |
| 79MA (81) | n | n | n | n/n |
| 80MA (81) | n | n | n | R/n |
| 81MA (81) | n (1.4) | n | n | R/n |
| 115MA (82) | nd | nd | n | nd |
| 126MA (88) | n | n | n | n/R |
| 130MA (88) | r | r | n | nd |
| 121MA (119) | n (0.6) | r | n | nd |
| 122MA (119) | n (1.2) | r | n | nd |
| 127MA (338) | n | n | n | nd |
| 128MA (348) | n | n | n | nd |
| 107MA (353) | r (1.0) | n | n | nd |

[a]n, within normal range; r, reduced response, values in parenthesis show the percentage by which marginal cases (within 2% of the lower limit of the normal range) fall within the given class. [b]n, within normal range; R, resistant; nd, not determined.

### Effect of *TP53* mutations on $G_1$ arrest

Among the mutation-carrying strains, those with mis-sense mutations in the DNA binding region of p53 all showed reduced $G_1$ arrest, although this was marginal for two strains of family 85 (109MA, 110MA) with a mutation in codon 180. Two strains heterozygous for splice site mutations (178MA, 191MA) gave responses within the normal range. This was probably not due simply to reduced gene dosage as a consequence of truncation of the product of the mutated alleles, because the stop codon mutation in strain 193MA resulted in a reduced response.

### Loss of the wt p53 allele drastically reduces $G_1$ arrest

Two strains (161MA-F, 172MA) that had lost the wt p53 allele (mut/–) but retained mutations in codons 344 and 337, respectively, showed no arrest in the transient assay and greatly reduced arrest when measured at late times in the permanent assay. This confirms a similar finding in MDAH087, homozygous for a mutation in codon 248 (Dulic et al, 1994; Tainsky et al, 1995). Mutations in codons 344 and 337 affect the oligomerization of p53 (Davison et al, 1998). In vitro L344P prevents dimerization and binding to the p53 DNA consensus sequence, whereas R337C has

 *© Cancer Research Campaign 1999*

**Table 4** Concordance between $G_1$ arrest and clonogenic radiosensitivity

| Group | Number of strains | $G_1$ arrest | Radiation survival | |
|---|---|---|---|---|
| | | | Normal | Resistant |
| LF mut/wt | 10 | Normal | 2 | 1 |
| | | Reduced | 1 | 6 |
| LF mut/- | 2 | Normal | 0 | 0 |
| | | Reduced | 2 | 0 |
| LF non-mutation | 6 | Normal | 3 | 3 |
| | | Reduced | 0 | 0 |

a lowered thermal stability such that tetramer formation and hetero-oligomerization with wt p53 are greatly impaired at physiological temperatures. Since tetramerization is required for transactivation of p53 target genes, the reduced $G_1$ arrest seen in 161MA-F and 172MA is consistent with a failure to induce p21$^{WAF1/CIP1}$.

### $G_1$ arrest is not defective in LF families without *TP53* mutations

A major observation of this study was the finding that strains from all nine families without *TP53* mutations (Group 4, Table 3) showed normal checkpoint responses in at least two, and usually all, of the three conditions assayed. Direct sequencing of DNA from normal tissue from donors of these cells had failed to detect any *TP53* mutations (Varley et al, 1997) but it was possible that the fibroblast cultures derived from these donors might have acquired mutations in vitro, as had been observed previously (Mirzayans et al, 1995). To check this possibility we tested cells at passage numbers comparable to those used in the $G_1$ arrest experiments using the FASAY (Table 1) which permits detection of expressed wt and mis-sense mutant p53 alleles (Flaman et al, 1995; Lomax et al, 1997). As expected, germline mis-sense mutations yielded about 50% mutant colonies in the yeast functional assay, whereas the splice site mutation in 178MA yielded 3% mutant colonies, which was within the background frequency of ≤ 5% obtained with wild-type normal cells. Most of the strains from families without *TP53* mutations gave mutant colonies at frequencies within, or close to, the normal range. However, 80MA and 128MA gave increased numbers of mutant colonies with some cDNA preparations but not others, the reason for which is unknown. The increased number of mutant colonies did not appear to be associated with reduced $G_1$ arrest since both strains showed normal $G_1$ arrest.

### $G_1$ arrest is not a consistent predictor of clonogenic radiation resistance in LF fibroblasts

In a previous study (Williams et al, 1997) we demonstrated an inverse correlation between the extent of $G_1$ arrest and radiosensitivity measured after exposure to ionizing radiation given at either high- or low-dose rates. Because exposure to low-dose rate radiation provides greater discrimination of sensitive and resistant cell populations in the clonogenic assay, we used this method to expand the number of strains studied. Our original results contrasted cells from normal individuals which had, by definition, normal $G_1$ arrest and radiosensitivity, with Li-Fraumeni cells mainly from mutation carriers, which had reduced $G_1$ arrest and were radioresistant. However, in strains that had lost the wild-type allele (172MA, R337C/- and 161MA-F, L344P/-) there was reduced $G_1$ arrest but normal radiosensitivity, clearly showing that the two events are dissociated in the absence of intact p53 (Table 3). The lack of $G_1$ arrest in p53 mut/– strains mimics that of AT homozygotes (Nagasawa et al, 1985), which are deficient in p53 stabilization following irradiation (Kastan et al, 1992). A similar effect is seen in embryo (Westphal et al, 1997) and adult skin (JMB and MJG, unpublished observations) fibroblasts from ATM and p53 knockout mice. However, AT homozygotes are more sensitive to ionizing radiation whereas p53 mut/– cells have normal radiosensitivity (Boyle et al, 1998).

A caveat affecting the interpretation of the results is that our ability to make a proper classification is critically dependent on the number of strains used to define normal limits for each of the parameters studied, because the group mean will be less strongly influenced by outliers as the sample size increases. The present sample size ($n$ = 12) is a compromise that allowed a reasonable accuracy with the available material and manpower resources. Nevertheless, these observations do appear to emphasize an apparent variability in phenotypes among Li-Fraumeni cell strains which may be controlled in part by the site of *TP53* mutation and by the genetic background in which it is expressed. Where a mutation produces a strong effect (e.g. R248Q in family 266, Table 3; R175H in family 86, Boyle et al, 1998) cells from different members of the family have similar phenotypes that are sufficiently different from wild-type to place them in different categories. However, where a mutation has a weaker effect, the phenotypes may be close to wild-type and small differences in genetic background may place different individuals into different categories. This is illustrated by family 85 (E180K) where strains 109MA and 110MA both showed normal $G_1$ arrest, but 110MA was slightly more resistant than 109MA and appeared discordant in comparison.

### Cellular phenotypes and p53 involvement in non-mutation families

The nine families without *TP53* mutations included both classical LFS (five families) and LFL (four families) patterns of disease. Within these families the most likely individuals to be carrying a genetic predisposition towards cancer are those affected by cancer. The majority of strains (12 of 16) used were from affected individuals, so it is highly probable that we are mainly looking at strains carrying genetic predisposition towards cancer. Our results provide no clear evidence to suggest that defective p53 expression is the cause of cancer predisposition in these families.

Recently, a number of genes have been identified that have homology with p53, e.g. p73, p53CP and KET (Bian and Sun, 1997; Jost et al, 1997; Kaghad et al, 1997; Schmale and Bamberger, 1997). Superficially, p73 is an attractive candidate since, like p53, it is a sequence-specific transactivator that probably requires oligomerization, and overexpression of p73 induces p21 and blocks cell proliferation (Kaghad et al, 1997). But, unlike p53, p73 is neither stabilized nor activated by DNA damage although in other ways it fulfills many of the functions of p53 (Oren, 1997). Expression of p73 and KET appear largely restricted to neurological and epithelial tissues, respectively. Thus, at present, there may be a possibility that cancer predisposition in p53 non-mutation families could result from mutations in these genes, or in other homologues still to be discovered, in which case

© Cancer Research Campaign 1999 

cells from these families might be expected to show normal $G_1$ arrest due to an intact p53 response to DNA damage, such as we have observed in these families.

### ACKNOWLEDGEMENTS

We thank Mike Hughes and Jeff Barry for FACS analyses; Martine Lomax and Paula Duddy for performing the FASAY tests and Dr David Scott for critical reading of the manuscript. This study was funded by the UK Cancer Research Campaign.

### REFERENCES

Barnes DM, Hanby AM, Gillett CE, Mohammed S, Hodgson S, Bobrow LG, Leigh IM, Purkis T, MacGeoch C, Spurr NK, Bartek J, Vojtesek B, Picksley SM and Lane DP (1992) Abnormal expression of wild-type p53 protein in normal cells of a cancer family patient. *Lancet* **340**: 259–263

Bian J and Sun Y (1997) p53CP, a putative p53 competing protein that specifically binds to the consensus p53 DNA binding sites: a third member of the p53 family? *Proc Natl Acad Sci USA* **94**: 14753–14758

Birch JM, Hartley AL, Tricker KJ, Prosser J, Condie A, Kelsey AM, Harris M, Morris Jones PH, Binchy A, Crowther D, Craft AW, Eden OB, Evans GR, Thompson E, Mann JR, Martin J, Mitchell ELD and Santibanez-Koref MF (1994) Prevalence and diversity of constitutional mutations in the *p53* gene among 21 Li-Fraumeni families. *Cancer Res* **54**: 1298–1304

Boyle JM, Mitchell EL, Greaves MJ, Roberts SA, Tricker K, Burt E, Varley JM, Birch JM and Scott D (1998) Chromosome instability is a predominant trait of fibroblasts from Li-Fraumeni families. *Br J Cancer* **77**: 2181–2192

Davison TS, Yin P, Nie E, Kay C and Arrowsmith CH (1998) Characterisation of the oligomerisation defects of two p53 mutants found in families with Li-Fraumeni and Li-Fraumeni-like syndrome. *Oncogene* **6**: 651–656

DiLeonardo A, Linke SP, Clarkin K and Wahl GM (1994) DNA damage triggers a prolonged p53-dependent G1 arrest and long-term induction of Cip1 in normal human fibroblasts. *Genes Dev* **8**: 2540–2551

Dulic V, Kaufmann WK, Wilson SJ, Tlsty TD, Lees E, Harper JW, Elledge SJ and Reed SI (1994) p53-Dependent inhibition of cyclin-dependent kinase activities in human fibroblasts during radiation-induced G1 arrest. *Cell* **76**: 1013–1023

Flaman J-M, Frebourg T, Morea V, Charbonnier F, Martin C, Chappuis P, Sappino A-P, Limacher J-M, Bron L, Benhattar J, Tada M, Van Meir EG, Estreicher A and Iggo RD (1995) A simple p53 functional assay for screening cell lines, blood and tumours. *Proc Natl Acad Sci USA* **92**: 3963–3967

Jost CA, Marin MC and Kaelin WG (1997) p73 is a human p53-related protein that can induce apoptosis. *Nature* **389**: 191–194

Kaghad, Bonnet H, Yang Y, Creancier L, Biscan J-C, Valent A, Minty A, Chalon P, Lelias J-M, Dumont X, Ferrara P, McKeon F and Caput D (1997) Monoallelically expressed gene related to p53 at 1p36, a region frequently deleted in neuroblastoma and other human cancers. *Cell* **90**: 809–819

Kastan MB, Onyewere O, Sidransky D, Vogelstein B, Lu S and Weinstein IB (1991) Participation of p53 protein in cellular responses to DNA damage. *Cancer Res* **51**: 6304–6311

Kastan MB, Zhan Q, El-Deiry WS, Carrier F, Jacks T, Walsh WV, Plunkett BS, Vogelstein B and Fornace AJ Jr (1992) A mammalian cell cycle checkpoint pathway utilising p53 and GADD45 is defective in ataxia-telangiectasia. *Cell* **71**: 587–597

Kuerbitz SJ, Plunkett BS, Walsh WV and Kastan MB (1992) Wild-type p53 is a cell cycle checkpoint determinant following irradiation. *Proc Natl Acad Sci USA* **89**: 7491–7495

Li FP and Fraumeni JF Jr (1969) Soft tissue sarcomas, breast cancer, and other neoplasms: a familial syndrome? *Ann Intern Med* **71**: 747–752

Li FP, Fraumeni JF, Mulvihill JJ, Blattner WA, Dreyfus MG, Tucker MA and Miller RW (1988) A cancer family syndrome in twenty-four kindreds. *Cancer Res* **48**: 5358–5362

Li Y-C, Nagasawa H, Dahlberg WK and Little JB (1995) Diminished capacity for p53 in mediating a radiation-induced G1 arrest in established human tumor cell lines. *Oncogene* **11**: 1885–1892

Little JB (1968) Delayed induction of DNA synthesis in irradiated human diploid cells. *Nature* **218**: 1064–1065

Little JB (1970) Irradiation of primary human amnion cell cultures: effects on DNA synthesis and progression through the cell cycle. *Radiation Res* **44**: 674–699

Little JB and Nagasawa H (1985) Effect of confluent holding on potentially lethal damage repair, cell cycle progression, and chromosomal aberrations in human normal and ataxia-telangiectasia fibroblasts. *Radiation Res* **101**: 81–93

Lomax ME, Barnes DM, Gilchrist R, Picksley SM, Varley JM and Camplejohn RS (1997) Two functional assays employed to detect an unusual mutation in the oligomerisation domain of p53 in a Li-Fraumeni like family. *Oncogene* **14**: 1869–1874

Mirzayans R, Aubin RA, Bosnich W, Blattner WA and Paterson MC (1995) Abnormal pattern of post-γ-ray DNA replication in radioresistant fibroblast strains from affected members of a cancer-prone family with Li-Fraumeni syndrome. *Br J Cancer* **71**: 1221–1230

Nagasawa H, Latt SA, Lalande ME and Little JB (1985) Effects of X-irradiation on cell cycle progression, induction of chromosomal aberrations and cell killing in ataxia telangiectasia (AT) fibroblasts. *Mutation Res* **148**: 71–82

Oren M (1997) Lonely no more: p53 finds its kin in a tumour suppressor haven. *Cell* **90**: 829–832

Schmale H and Bamberger C (1997) A novel protein with strong homology to the tumor suppressor p53. *Oncogene* **15**: 1363–1367

Tainsky MA, Bischoff FZ and Strong LC (1995) Genomic instability due to germline p53 mutations drives preneoplastic progression toward cancer in human cells. *Cancer Metastasis Rev* **14**: 43–48

Varley JM, Chapman P, McGown G, Thorncroft M, White GRM, Greaves MJ, Scott D, Speadborough A, Tricker KJ, Birch JM, Evans DGR, Reddel R, Camplejohn RS, Burn J and Boyle JM (1998) Genetic and functional studies of a germline *TP53* splicing mutation in a Li-Fraumeni family. *Oncogene* **16**: 3291–3298

Varley JM, McGown G, Thorncroft M, Santibanez-Koref MF, Kelsey AM, Tricker KJ, Evans DGR and Birch JM (1997) Germline mutations of *TP53* in Li-Fraumeni families: an extended study of 39 families. *Cancer Res* **57**: 3245–3252

Westphal CH, Schmaltz C, Rowan S, Elson A, Fisher DA and Leder P (1997) Genetic interactions between atm and p53 influence cellular proliferation and irradiation-induced cell cycle checkpoints. *Cancer Res* **57**: 1664–1667

Williams KJ, Boyle JM, Birch JM, Norton JD and Scott D (1997) Cell cycle arrest defect in Li-Fraumeni syndrome: a mechanism of cancer predisposition? *Oncogene* **14**: 277–282

*© Cancer Research Campaign 1999*
